# Probiotic Bacterial Application in Pediatric Critical Illness as Coadjuvants of Therapy

**DOI:** 10.3390/medicina57080781

**Published:** 2021-07-30

**Authors:** Christian Zanza, Tatsiana Romenskaya, Yaroslava Longhitano, Fabio Piccolella, Fabrizio Racca, Michele Fidel Tassi, Francesca Rubulotta, Ludovico Abenavoli, Dana Shiffer, Francesco Franceschi, Alessio Migneco, Angela Saviano, Andrea Piccioni, Veronica Ojetti

**Affiliations:** 1 Department of Emergency Medicine, Policlinico Gemelli/IRCCS- University of Catholic of Sacred Heart, 00168 Rome, Italy; francesco.franceschi@unicatt.it (F.F.); alessio.migneco@policlinicogemelli.it (A.M.); saviange@libero.it (A.S.); andrea.piccioni@policlinicogemelli.it (A.P.); veronica.ojetti@unicatt.it (V.O.); 2 Foundation “Ospedale Alba-Bra” Michele and Pietro Ferrero Hospital, 12060 Verduno, Italy; lon.yaro@gmail.com; 3 Department of Emergency Medicine, Anesthesia and Critical Care Medicine, Michele and Pietro Ferrero Hospital, 12060 Verduno, Italy; 4 Department of Anesthesia and Critical Care Medicine, St. Antonio and Biagio and Cesare Arrigo Hospital, 15121 Alessandria, Italy; tatsiana_romenskaya@yahoo.it (T.R.); fpiccolella@ospedale.al.it (F.P.); fracca@ospedale.al.it (F.R.); 5 Department of Emergency Medicine, St. Antonio and Biagio and Cesare Arrigo Hospital, 15121 Alessandria, Italy; michelefidel.tassi@gmail.com; 6 Department of Anaesthesia and Intensive Care Medicine, Imperial College London, London SW7 2AZ, UK; frubulotta@hotmail.com; 7 Department of Health Sciences, Magna Graecia University, 88100 Catanzaro, Italy; l.abenavoli@unicz.it; 8 Department of Internal Medicine, Humanitas Clinical and Research Center, IRCCS, Humanitas University, 0089 Rozzano, Italy; dana.shiffer@humanitas.it

**Keywords:** pediatric critical illness, pediatric intensive care unit, children, probiotic, lactobacillus, lactobacillus reuteri

## Abstract

The use of probiotics in critically ill adult and children patients has been growing exponentially over the last 20 years. Numerous factors in pediatriac intensive care unit (PICU) patients may contribute to intestinal dysbiosis, which subsequently promotes the pathobiota’s growth. Currently, lactobacillus and bifidobacterium species are mainly used to prevent the development of systemic diseases due to the subverted microbiome, followed by *streptococcus*, *enterococcus*, *propionibacterium*, *bacillus* and *Escherichia coli*, *Lactobacillus rhamnosus GG*, and *Lactobacillus reuteri DSM 17938*. The aim of this article is to review the scientific literature for further confirmation of the importance of the usage of probiotics in intensive care unit (ICU) patients, especially in the pediatric population. A progressive increase in nosocomial infections, especially nosocomial bloodstream infections, has been observed over the last 30 years. The World Health Organization (WHO) reported that the incidence of nosocomial infections in PICUs was still high and ranged between 5% and 10%. Petrof et al. was one of the first to demonstrate the efficacy of probiotics for preventing systemic diseases in ICU patients. Recently, however, the use of probiotics with different lactobacillus spp. has been shown to cause a decrease of pro-inflammatory cytokines and an increase in anti-inflammatory cytokines. In addition, in some studies, the use of probiotics, in particular the mix of Lactobacillus and Bifidobacterium reduces the incidence of ventilator-associated pneumonia (VAP) in PICU patients requiring mechanical ventilation. In abdominal infections, there is no doubt at all about the usefulness of using Lactobacillus spp probiotics, which help to treat ICU-acquired diarrhoea episodes as well as in positive blood culture for candida spp. Despite the importance of using probiotics being supported by various studies, their use is not yet part of the standard protocols to which all doctors must adhere. In the meantime, while waiting for protocols to be drawn up as soon as possible for use in PICUs, routine use could certainly stimulate the intestine’s immune defences. Though it is still too early to say, they could be considered the drugs of the future.

## 1. Introduction

Interest in the use of probiotics in critically ill patients in ICUs for adults or children has been growing exponentially over the last 20 years with numerous studies each year, but the first trials on the importance of probiotics were carried out about 30 years ago [1].

In 2002, for the first time, the World Health Organization (WHO) described probiotics as live micro-organisms that, when administered in the necessary quantities, help to maintain the homeostasis of the intestinal flora [2]. In fact, the microbiome is a collection of microorganisms that live in symbiosis with the human body and play a crucial role in regulating the response of the intestinal immune system through production of anti-inflammatory cytokines and inhibition of pro-inflammatory cytokines [3,4,5,6].

In addition, the microbiota builds a physical barrier between the outside and inside of our bodies with the help of the caliciform cells, which produce mucus made up of proteins that strengthen the barrier of the intestinal wall [7,8]. Moreover, the enterocytes produce antibacterial substances, such as bacteriocins and lactate, that can inhibit the growth of patho-biota [9,10]. Studies conducted in the past have shown that the loss of normal intestinal flora and its replacement by the growth of pathogenic bacteria (dysbiosis) can lead to the development of critical illnesses [11]. Sepsis has an important impact on the gastrointestinal function and the associated permeability alteration can become a source of systemic infection [12].

The composition of the gut microbiome in ICU patients has previously been shown to play a major role in determining the outcome in those patients. Additionally, enteral nutrition and the use of various drugs, particularly antibiotics, can lead to alteration of the gut microbiome in ICU patients [13,14,15].

Regrettably, despite this solid evidence, probiotics are not part of the standard protocols in the ICU [16].

The most commonly used probiotics are lactobacillus and bifidobacterium species, followed by streptococcus, enterococcus, propionibacterium, bacillus, and *Escherichia coli* [17]. In particular, *Lactobacillus rhamnosus GG* [18] and *Limosilactobacillus reuteri* [19] have been widely used in the treatment of infections of the gastrointestinal tract, inflammatory diseases, and drug-induced diarrhoea in the ICU in both adults and children. In addition to those, probiotics based on certain yeast species, such as *Saccharomyces boulardii* [20] and *Saccharomyces cerevisiae* [21,22], are also widely used, especially in the treatment of diseases of the gastrointestinal tract [20,22].

## 2. Materials and Methods

A literature search was performed using the following databases to identify relevant studies in indexed scientific journals: Pubmed, MEDLINE (via Ovid), EMBASE (via Ovid), and the Cochrane Controlled Clinical trials register, using the following terms: pediatric critical illness, pediatric intensive care unit, children, probiotic, *Lactobacillus*, *Lactobacillus reuteri* with filters for humans, language (English), and time of publication (1 January 1991 to 28 February 2021). We excluded editorials, commentaries, letters to the editor, opinion articles, meeting abstracts, and original articles lacking an abstract.

Research was limited to clinical trials, meta-analysis, randomized controlled trials (RCT), review, and systematic review. Search criteria included the following: “probiotics and pediatric intensive care”, “lactobacillus and pediatric intensive care”, “lactobacillus reuterii and pediatric intensive care”, “probiotics and pediatric critical illness”, “lactobacillus and pediatric critical illness”, “lactobacillus reuterii and pediatric critical illness”, “probiotics and children”, “lactobacillus and children”, and “lactobacillus reuterii and children”.

### 2.1. Probiotics in the ICU

A progressive increase in nosocomial infections, especially nosocomial bloodstream infections, has been observed over the last 30 years. In 2007, Singhi et al. studied the incidence of nosocomial infections in pediatric patients in intensive care units in India. They observed a total of three periods and found that the frequency of nosocomial infections was 32.6% in the years 1991–1996, 33.1% in 1999–2001, and 33.6% in 2002–2003. The total number of patient days per year during the three time periods was 2589, 2029, and 2176, respectively, translating to 3.63, 5.94, and 4.99 episodes of nosocomial bloodstream infection per 100 patient-days, respectively. Furthermore, Singhi et al. concluded that the data obtained were comparable to the results obtained from studies in developing countries, and it was also observed that, in these countries, the course of nosocomial bloodstream infection in PICU persisted in a more severe form [23]. In 2011, the WHO reported that the incidence of nosocomial infections in PICUs was still high and ranged between 5% and 10%.

In 2012, Petrof et al. conducted a systematic review where he highlighted the importance of using probiotics in preventing systemic diseases in ICU patients, showing that the association of probiotics with the conventional prescribed therapy set in the ICU leads to a reduction in complications related to infections: 11 trials (RR 0.82; 95% CI 0.69–0.99; *p* = 0.03; test for heterogeneity *p* = 0.05; I2 44% [24].

Several studies in the 1990s and early 2000s demonstrated the efficacy of *Lactobacillus rhamnosus GG*, which was first isolated by human intestinal flora, and administration of this strain has shown efficacy in improving symptoms caused by rotavirus [25,26] in pediatric patients, to be able to modulate systemic immune responses, such as gastroenteritis [27], and to prevent atopic manifestations in this type of patient [28].

A randomized, double-blind, placebo-controlled trial was conducted between November 2014 and October 2015 in ICUs in India. Angurana S. K. et al., who has long been active in research on microbiota and probiotics and their contribution in the development of systemic diseases, decided to analyze the effect of probiotics on the level of cytokines in children 3 months to 12 years old with severe sepsis (probiotic group *n* = 50 vs. placebo group *n* = 50). The result was extraordinary: on day 7, the probiotics group receiving VSL#3 contained *Lactobacillus paracasei, L. plantarum, L. acidophilus, L. delbrueckii, Bifidobacterium longum, B. infantis, B. breve, Streptococcus salium, B. infantis and B. delbrueckii. breve,* and *Streptococcus salivarius*, and had a statistically significant decrease of proinflammatory cytokines: IL6 *p* = 0.001; IL 12p70 *p* = 0.001; IL 17 *p* = 0.01; and TNF-α *p* = 0.01; and a statistically significant increase of anti-inflammatory cytokines: IL 10 *p* = 0.02 and TGF-β1 *p* = 0.01 [29].

In 2016, Wang Y. et al. [30] published a meta-analysis analyzing twenty-three trials with a total of 6269 children in the PICU on the effect of probiotic use in the preventing and treating of respiratory tract infections in PICU patients. With regard to the trials analyzed that reported the occurrence of at least one episode of respiratory tract infection, it was seen that the ‘probiotics’ group had a statistically significantly lower probability of developing this complication (RR 0.89, CI 95% 0.82–0.96, *p* = 0.004). Another statistically significant result was obtained analyzing six trials including 2067 ICU children: the days of RTIs were analyzed and it was observed that the ‘placebo’ group had more sick days than the ‘probiotics’ group (weighted MD 0.16, CI 95% 0.29–0.02, *p* = 0.03).

### 2.2. Respiratory Tract Infection and Probiotics

Respiratory tract infections in children of different age groups represent a major problem that requires attention because it is a main cause of morbidity and mortality worldwide [31,32].

These infections may have different etiologies (viral, bacterial, or fungal), thus inappropriate therapy for the etiological agent, for example, administration of antibiotics in the case of a viral infection, could have a knock-on effect on the intestinal microflora, altering the balance of the microbiota and promoting pathobiota overgrowth [33].

Often, patients admitted to the ICU are sedated and intubated with the oral-tracheal tube becoming a bridge between the oral cavity and the pulmonary “ecosystem”, and migration of bacteria can occur more easily [34]. This migration is also facilitated by reduced mucociliary clearance decreased cough reflex [35]. Another important risk factor of contiguous infection [36] is micro-aspiration [37] of oral flora; this subverting [38] of normal oral flora promotes its substitution by pathogenic bacteria, such as *Pseudomonas aeruginosa* and *Klebsiella pneumoniae* [39].

In addition, when an inflammatory disease (for example, ARDS, HAP, and VAP) affects the respiratory system, there will be a decrease in pulmonary surfactant; this leads to the reduction of bacteria susceptible to surfactant and can promote pathobiome growth [40,41,42].

Recently, Banupriya et al. [43] conducted a study in the period between November 2011 and July 2013, in which 150 pediatric patients requiring mechanical ventilation for more than 48 h in the intensive care unit were recruited. The pediatric patients were divided into two groups: the “probiotics” group (*n* = 75), which received a mix of *Lactobacillus acidophilus*,* L. rhamnosus, Lactobacillus plantarum*,* L. casei*,* Lactobacillus bulgaricus*,* Bifidobacterium longum*,* B. infantis*,* Bifidobacterium breve*, and *Streptococcus thermophilus* for 7 days or until discharge; and the “control” group (*n* = 75), which was not treated with the above cocktail of probiotics. The result obtained plays an important role in the indications for the use of probiotics in this type of population; a statistically significant decrease in the incidence of VAP was observed in the probiotics group (17.1% vs. 48.6%, *p* < 0.001). The VAP rates were also significantly lower in the probiotics group compared with the control group (22 per 1000 ventilated days vs. 39 per 1000 ventilated days, *p* = 0.02).

Mean duration of ICU stay in the probiotics group was 7.7 days compared with 12.54 days in the control group (*p* < 0.001). Mean duration of hospital stay was 13.13 days in the probiotics group and 19.17 days in the control group (*p* = 0.001). There was a decrease in trend of mortality due to VAP in the patients of the probiotics group, although it was not statistically significant (1.4% vs. 4.2%, *p* = 0.641). In addition, the probiotics group had lower colonization rates with potentially pathogenic organisms (*Klebsiella and Pseudomonas*) (34.3% versus 51.4%; *p* = 0.058) and reductions of VAP caused by *Klebsiella* (4.2% versus 19.4%, *p* = 0.01) and *Pseudomonas* (4.2% versus 16.7%, *p* = 0.03). Complications related to the administration of probiotics in patients were not observed during the study period.

In 2018, Shimizu et al. showed that, during ICU stays, the daily symbiotics administration (in particular, bifidobacterium breve strain yakult, lactobacillus casei strain Shirota, and galacto-oligosaccharides) was very useful to prevent VAP. This result was obtained in the 35 patients treated with symbiotics and compared with the result of the 37 ICU patients group not treated with symbiotics. The incidence of VAP was significantly lower in the synbiotics than the no-synbiotics group (14.3% vs. 48.6%; *p* < 0.05) [44]. The incidence of enteritis was also significantly lower in the synbiotics group compared with the no-synbiotics group (6.3% vs. 27.0%; *p* < 0.05).

### 2.3. Abdominal Infection and Probiotics

In pediatric patients in the ICU, it is very common to find diarrhoea, mainly associated with the use of antibiotics after the exclusion of other possible etiologies [45]. However, the use of probiotics helps to restore the altered homeostasis of the intestinal flora and microbiome. Szajewska H. et al. [46] reported five RCT studies with a total of 445 children recruited and concluded that the incidence of ICU-acquired diarrhoea episodes decreased in children treated with *Lactobacillus rhamnosus GG* from 23% to 9.6% (RR 0.48, CI 95% 0.26–0.89) and from 20.9% to 8.8% in 1653 children treated by *S. boulardii* (RR 0.43, CI 95% 0.30–0.60 from six RCTs). Szajewska H. and her research group also conducted a meta-analysis [18] in 2013, analyzing the results of 11 RCTs study involving 2444 children, and showed that the use of *Lactobacillus rhamnosus GG* probiotics reduced the days of persistent diarrhoea in children (mean difference −1.05 days, 95% CI −1.7 to −0.4) and the result from the European studies was even more positive in terms of duration of diarrhoea episodes (mean difference −1.3 days, 95% CI −2.0 to −0.5).

### 2.4. Candida spp. Infection and Probiotics

There are about 10–20% of patients in the ICUs who have positive blood cultures for Candida spp. during hospitalization [47].

A more recent epidemiological study conducted in Italian ICUs was sponsored by the European Confederation of Medical Mycology (ECMM) in collaboration with the study group of the Italian Federation of Human and Animal Mycology (FIMUA). Crude mortality reached 50% with a marked difference between the two different types of hospitalized patients—61% in medical patients and 46% in surgical patients [48,49]. Unfortunately, the incidence of candidemia has dramatically increased in the past three decades [50,51].

Kumar S et al. conducted a prospective double-blinded randomized controlled trial from November 2007 to October 2008 to investigate the protective effect of probiotics in the prevention of Candida colonization in children admitted to the pediatric ICU. In total, 150 children aged between 3 months and 12 years were recruited and randomized into two identical groups: placebo group (*n* = 75) and probiotics group (*n* = 75). Probiotics contained *Lactobacillus acidophillus*,* Lactobacillus rhamnosum*,* Bifidobacterium longum*,* Bifidobacterium bifidum*,* Saccharomyces boulardi*, and *Streptococcus thermophilus*. The researchers demonstrated that, at day 14 the probiotics group developed less candida colonization (31.4% of children of probiotics group vs. 50% of placebo group, RR 0.63; 95% CI 0.41–096; *p* = 0.02). Furthermore, the number of patients colonized during the study period was statistically lower in the probiotics group than in the placebo group (42.6% vs. 60%, *p* = 0.03). In patients who did not receive probiotics, a statistically significant pathological growth of candida was observed on the rectal swab (37% vs. 26% of probiotics group, RR 0.71, 95% CI 0.53–0.94; *p* = 0.01). Candiduria was significantly less common in the probiotic group than in the placebo group (17.3% vs. 37.3%; relative risk 0.46; 95% confidence interval 0.26–0.82; *p* = 0.006) [52,53].

### 2.5. Probiotics and Recent Guidance in Children with Selected Clinical Conditions

On reviewing the scientific literature available on major search engines, it was noted that, even until recently, there has been a lack of detailed guidelines on the use of probiotics in ICU patients. In 2018, guidance on the use of probiotics in clinical practice for selected clinical conditions and for specific vulnerable groups in PICU was published [54].

Despite numerous studies published in the past, the current guidance does not provide information on the use of probiotics in the prevention of common infections in children attending day care centers. However, the use of probiotics, in particular, *Lactobacillus rhamnosus GG (LGG)*, outside the PICU can effectively reduce the risk of upper respiratory tract infections. This was confirmed by Hojack I. et al. in 2017, conducting an RCT that analyzed LGG (*n* = 742) at the dose of 10^9^ CFU and found a reduction in the risk of upper respiratory tract infection [55].

The authors of the Guidance [54] recommend, in order to prevent an antibiotic-associated diarrhoea in children, using *Lactobacillus rhamnosus GG (LGG)* or *S. boulardii*, the latter of which has been particularly effective in preventing systemic C. difficile infection. Indeed, probiotics should ideally be initiated early after the first diarrhoeal discharge.

### 2.6. Safety of Probiotics

Probiotics tend to be safe, but there are concerns about their use in debilitated and immunosuppressed patients owing to the risk of causing sepsis related to probiotic use [56].

*L. rhamnosus* normally lives in the oral, rectal, and vaginal flora, but cases of liver abscess and endocarditis have been described with this bacterium as the main aetiological agent [57,58,59,60].

Kunz et al. [61] described two cases of bacteraemia after *Lactobacillus GG* supplementation in premature infants with short bowel syndrome and fed via gastrostomy and jejunostomy. These two cases described aroused the interest of the scientific world and, in 2004, Rosemary J.Y. [62] set out his theory of translocation of resident intestinal bacteria that led to sepsis in those two premature babies. Egervarn M. et al. [63] conducted a study in 2009 with the aim of discovering the cause of antibiotic resistance of most pathogenic bacteria of the intestinal flora caused by probiotics. This resistance is apparently due to the genetic transfer of, in particular, the tet (M) and tet (S) genes from various lactobacillus species and the tet (W) gene from various species of Bifidobacterium.

Land et al., in 2005, also described two cases of probiotics—sepsis in 4-month-old and 6-year-old children treated with *Lactobacillus GG* [64]. These children had a remote pathological history positive for immunodeficiency conditions before being treated with probiotics.

Interestingly, in 2004, Honeycutt et al. conducted a randomized, double-blinded, placebo-controlled trial involving 61 pediatric ICU patients. The result went against the conclusions of many studies conducted in the past. Honeycutt TC et al. found a non-statistically significant trend of developing a nosocomial infection: six patients who received treatment with *Lactobacillus GG* developed 11 nosocomial infections (RR 1.94, CI95% 0.53–7.04; *p* = 0.31) [65].

However, in another 10-year study conducted in Finland by Saminen MK et al., no statistically significant trend of the lactobacillus bacteremia isolates among all blood cultures (*p* = 0.9702) or the proportion of Lactobacillus isolates among all positive blood cultures (*p* = 0.7282) was observed [66]. The major limitation of the study was the subjects recruited: most of the participants using probiotics were in good health.

Manzoni et al. [67] conducted a retrospective study by reviewing the medical records of preterm infants admitted in the years 2003–2008 in two large tertiary NICUs in northern Italy. Preterm infants were treated with *Lactobacillus GG* in a single dose of 3 × 10^9^ CFU/day from the fourth day of life for 4–6 weeks. There were no side effects of probiotic use in this group of patients and no evidence of probiotic-related bacteremia and/or sepsis.

The result of the double-blinded, randomized, controlled clinical trial conducted by Srinivasan et al. [68] also confirmed the safety of probiotic use in children in PICU. They recruited 94 children between 1 and 3 years of age on mechanical ventilation requiring enteral feeding. These children were treated with the synbiotic mixture consisting of two probiotic strains, *Lactobacillus paracasei NCC 2461* and *Bifidobacterium longum NCC 3001*.

Analyzing the scientific literature available to date, we have not found any articles reporting fungemia nor septicaemia in immunocompromised and/or critically ill patients treated with S. boulardii probiotics [69,70].

## 3. Conclusions

The importance of using probiotics in the PICU is supported by various studies and their use is growing daily. Table 1 shows the most important studies supporting the use of probiotics mentioned in our article. Despite the scientific evidence, the use of probiotics in PICU patients is not yet part of the standard protocols. This is probably because, although they are safe and evidence confirms their importance in restoring the balance of the microbiota of pediatric and non-pediatric patients, and in assisting standard therapy in the course of even serious infectious diseases, these are the most fragile patients where the microbiota, although rarely, can induce bacteremia, fungemia, and sepsis. Well-designed multi-center RCTs are needed to address these issues before the routine use of probiotics is recommended in critically ill children.

## Figures and Tables

**Table 1:** Summary of high-power studies supporting the benefit of probiotics in the pediatriac intensive care unit (PICU).

	Authors	Study and Period	Patient Group	Administrations	Main Results
1	Singhi S. et al.	1991–1996, 1999–2000, 2002–2003 High statistical power	861 episodes of nosocomial bloodstream infection were documented in 841 patients	___	- Increase of frequency of nosocomial infection in the PICU - Increasing trend of resistance to the commonly used cephalosporins.
2	Petrof et al.	Sistemic review 1980–2011 High statistical power	23 randomized controlled trials enrolling critically ill adults, which evaluated probiotics compared with a placebo and reported clinically important outcomes	Probiotics with the conventional prescribed therapy set in the ICU leads	- Using probiotics in preventing systemic diseases leads to a reduction in complications related to infections.
3	Honeycutt TC et al.	Randomized, double-blind, placebo-controlled trial, April 2004–December 200 Low statistical power	61 total pediatric ICU patients: 31 of treatment group vs. 30 of placebo group	One capsule of Lactobacillus rhamnosus strain ones a day vs. one capsule of insulin once a day	- No results in support of the usage of probiotics.
4	Angurana SK et al.	Randomized, double-blind, placebo-controlled trial, November 2014–October 2015 High statistical power	100 children 3 months to 12 years old with severe sepsis in the ICUs (probiotic group *n* = 50 vs. placebo group *n* = 50)	Probiotic group received a multistrain, high-dose probiotic product VSL#3, which contained Lactobacillus paracasei, *L. plantarum, L. acidophilus, L. delbrueckii, Bifidobacterium longum, B. infantis, B. breve, Streptococcus salium, B. infantis and B. delbrueckii. breve,* and *Streptococcus salivarius*	- Using probiotics leads to a decrease of proinflammatory cytokines and an increase of anti-inflammatory cytokines.
5	Wang Y. et al.	Systematic review and meta-analysis, from the earliest available date to 30 April 2016. High statistical power	23 trials involving 6269 children in the PICUs, probiotics groups vs. placebo groups	___	- Probiotics lead to lower probability of developing complication and fast healing.
6	Banupriya et al.	Open-label randomized controlled trial, November 2011 and July 2013 High statistical power	150 pediatric patients requiring mechanical ventilation for more than 48 h in the PICU (75 vs. 75 patients)	Mix of Lactobacillus acidophilus, *L. rhamnosus, Lactobacillus plantarum*,* L. casei*,* Lactobacillus bulgaricus*,* Bifidobacterium longum*,* B. infantis*,* Bifidobacterium breve*, and *Streptococcus thermophilus* for 7 days or until discharge	- Probiotics lead to a decrease in the incidence of VAP and a decrease in the ICU stay; - Probiotics lead to lower colonisation by potentially pathogenic organisms, Klebsiella, and Pseudomonas.
7	Shimizu et al.	Randomized controlled trial, November 2011–September 2016 Intermediate statistical power	72 patients in the PICUs (35 patients receiving synbiotics and 37 patients not receiving synbiotics)	A daily symbiotics administration (in particular, bifidobacterium breve strain yakult, lactobacillus casei strain Shirota, and galacto-oligosaccharides).	- Probiotic lead to decrease of the incidence of VAP and of the incidence of enteritis.
8	Szajewska H. et al.	Recommendations, developed by the Working Group (WG) on Probiotics of the European Society for Pediatric Gastroenterology, Hepatology, and Nutrition, for the use of probiotics for the prevention of antibiotic-associated diarrhea (AAD) in children based on systematic review, 2016			- The incidence of ICU-acquired diarrhoea episodes decreased in children treated with Lactobacillus rhamnosus GG.
9	Kumar S. et al.	Prospective double-blinded, randomised controlled trial, November 2007–October 2008 High statistical power	150 PICU children aged between 3 months and 12 years: placebo group (*n* = 75) and probiotics group (*n* = 75)	Probiotics contained *Lactobacillus acidophillus, L. rhamnosum, Bifidobacterium longum, B. bifidum, Saccharomyces boulardi,* and *S. thermophilus*.	- Probiotics lead to less Candida colonisation and reduce a pathological growth of Candida; - Candiduria was less common in the probiotic group.
10	Manzoni et al.	Retrospective study, 2003–2008 Very high statistical power	743 VLBW infants	Lactobacillus GG as a single dose of 3 × 10^9^ CFU/day from the fourth day of life for 4 to 6 weeks	- Probiotics were well tolerated without any adverse effects and did not lead to bacteremia or sepsis episode attributable to Lactobacillus GG.
11	Simakachorn N. et al.	Controlled, double-blind, randomised clinical trial, August 2006–May 2009 Intermediate statistical power	94 patients between 1 and 3 years old under mechanical ventilation requiring enteral feeding	Synbiotic blend composed of two probiotic strains, *Lactobacillus paracasei* NCC 2461 and *Bifidobacterium longum* NCC 3001	- Probiotic’s formula was as well tolerated as the currently used formula and that it was safe.

High statistical power 0.8–0.9; intermediate statistical power 0.7–0.6; low statistical power < 0.5.

## Data Availability

Not applicable.
